# Investigation of the effects of oxidative stress, inflammation on the pathway of tryptophan/kynurenine in OCD

**DOI:** 10.1017/neu.2023.55

**Published:** 2023-11-28

**Authors:** Elif Delen, Cem Ismail Kucukali, Zerrin Karaaslan, Hande Yuceer, Seyma Punar, Mehmet Tolgahan Hakan, Ilhan Yaylim, Elif Ozkok

**Affiliations:** 1 Department of Neuroscience, Aziz Sancar Institute of Experimental Medicine, Istanbul University, Istanbul, Turkey; 2 Department of Genetics, Aziz Sancar Institute of Experimental Medicine, Istanbul University, Istanbul, Turkey; 3 Department of Molecular Medicine, Aziz Sancar Institute of Experimental Medicine, Istanbul University, Istanbul, Turkey

**Keywords:** Obsessive-compulsive disorder, oxidative stress, interferon gamma, tryptophan/kynurenine pathway, aryl hydrocarbon receptor, CYP1A1

## Abstract

**Objectives:**

Recent studies have shown that the distribution of the tryptophan/kynurenine pathway (KP) plays a role in the development of obsessive-compulsive disorder (OCD). We aimed to reveal the relationship between CYP1A1 rs464903 and aryl hydrocarbon receptor (AhR) rs10249788 associated with the KP and interferon gamma (IFN γ) and oxidative stress in OCD.

**Methods:**

In our study, the serum and DNAs of 150 samples, including 100 OCD patients and 50 controls, were used. The activity of glutathione peroxidase (GSH-Px), and the levels of IFN γ, thiobarbituric acid reactive substances (TBARS), tryptophan, and kynurenine were determined by biochemical methods. AhR rs10249788 and cytochrome P450 family CYP1A1 rs4646903, which interact directly with the KP, were analysed by polymerase chain reaction followed by restriction fragment length polymorphism. *P* < 0.05 was considered statistically significant.

**Result:**

There were no significant differences between groups in CYP1A1 rs4646903 and AhR rs10249788 while tryptophan and IFN γ were found to be higher in controls (*p* < 0.001, for both), and TBARS and indolamine-2,3-dioxygenase were found to be higher in OCD (*p* < 0.001, for both). There were significant correlations between IFN γ and TBARS and GSH-Px (*p* = 0.028, *p* = 0.020, respectively) in the OCD group.

**Conclusions:**

For the first time studied in OCD, it has been shown that IFN γ, tryptophan, oxidative stress parameters, and gene variants of CYP1A1 rs4646903 anAhR rs10249788 are shown effective on the KP.

Significant Outcomes

- It was determined that TBARS and IFN γ were effective on the kynurenine pathway in OCD.
- In the OCD patient group, TBARS and IDO activities were found to be increased and tryptophan levels were decreased in those carrying the CYP1A1 rs4646903 TT genotype; on the other hand, decreased IFN γ levels were detected in those carrying the TT, TC genotype.
- Increased TBARS and IDO activities in those carrying AhR rs10249788 CC and CT genotypes; while there was a decrease in tryptophan levels; IFN γ levels were found to be decreased in OCD patients with all three genotypes.

Limitations

- The limitation of our study can be considered that we could not reach more volunteers in OCD and control groups. Another limitation is the inability to investigate gene loci with other enzyme and protein levels that may have a role in KP due to the limited availability of resources. Concerning our study, it is aimed to study the genes expressing other enzymes involved in the serotonin, melatonin pathway, and KP related to OCD in future studies.

## Introduction

Obsessive-compulsive disorder (OCD) is a psychiatric disorder consisting of obsessions that cause anxiety and compulsions performed to relieve this anxiety (American Psychiatric Association, 2013).

OCD is the fourth most common psychiatric group after substance use, specific phobias, and major depression. OCD is a debilitating neuropsychiatric disorder with a lifetime prevalence of 2–3% and is estimated to be the 10th leading cause of disability in the World (Karno *et al*., 1988).

The aetiopathogenesis of OCD foresees the interaction of genetic and environmental factors (Pauls *et al*., 2014). The aetiological causes of OCD include an impaired immune system and its effects on serotonin, glutamate, and dopamine neurotransmitter levels as well as genetic and oxidative stress (Rodriguez *et al*., 2015; Marazziti *et al*., 2018).

A 1–5% of tryptophan, an essential amino acid, is metabolised to serotonin and melatonin, while 90–95% of it is oxidised to kynurenine by indolamine-2,3-dioxygenase (IDO), and then turns into kynurenic acid, anthranilic acid, and quinolinic acid molecules (Badawy, 2017). Since the production of serotonin is synthesised from tryptophan, it is in close relationship with the kynurenine pathway (KP) is the IDO, and the main regulatory molecule of this pathway is interferon gamma (IFN γ), a proinflammatory cytokine (Wang *et al*., 2015).

The main responsible transcription factor in the KP is the aryl hydrocarbon receptor (AhR). AhR interacts with the Hsp90 protein to form the AhR complex, and then the activated complex, AhR binds to the Aryl hydrocarbon receptor nuclear translocator (ARNT), which is called the nuclear translocator. As a result of the binding of the AhR-ARNT structure to the xenobiotic responsible element (XRE) in the promoters of target genes in the nucleus, interaction with CYP1A1 occurs (Anderson *et al*., 2013). The AhR gene is mapped to the 7p15 region on chromosome 7 and has a size of approximately 50 kb, consisting of 11 exons and 10 introns. There are studies showing that the polymorphic regions of the AhR gene have effects on the affinity of the AhR proteins and the activation of the signalling pathway (Micka *et al*., 1997).

The CYP1A1 gene belongs to Cytochrome P450 and is located on chromosome 15 (15q22-24). It has been known that binding of the AhR-ARNT complex is required for the transcriptional activation of the CYP1A1 gene (Anderson *et al*., 2013). Polymorphism studies on the CYP1A1 gene have been associated with various cancer groups such as lung, stomach, and squamous cancer (Wojtczak & Skretkowicz, 2009; Zhou *et al*., 2010).

Oxidative stress occurs when pro-oxidant molecules cannot be eliminated by antioxidant defence molecules in the organism, and thus, the balance is in favour of pro-oxidant molecules (Kar & Choudhury, 2016). In the literature, there are studies on the important roles of antioxidant molecules and free radicals in disease pathology in oxidative stress in OCD. In oxidative stress conditions, the effects of excessive reactive oxygen and nitrogen species cannot be reduced or eliminated with antioxidant defence enzymes and molecules. Brain tissue, which is very rich in lipid molecules, triggers the formation of lipid peroxidation reactions that occur as a chain and irreversible; in these reactions, disruption of the membrane structure leads to loss of neuron with the loss of cell integrity (Chakraborty *et al*., 2009; Behl *et al*., 2010; Madhura, 2015; Shrivastava *et al*., 2017; Maia *et al*., 2019; Mohammadi *et al*., 2021; Baratzadeh *et al*., 2021).

The KP metabolite balance is of great importance in maintaining brain functions (Vécsei *et al.*, 2013; Vamos *et al*., 2009). Studies in psychiatric diseases have shown that KP is one of the important sources of oxidative stress (O’Farrell & Harkin, 2015; Muneer, 2020).

The aim of our study was to reveal the relationship among polymorphisms of CYP1A1 rs4646903 and AhR rs10249788 associated with KP, the proinflammatory cytokine IFN γ, and the parameters of oxidative stress in OCD.

## Material and methods

### Study population

Our study was approved by the Istanbul University Istanbul Medical Faculty Clinical Research Ethic Committee (2020/125).

OCD patients were recorded among psychiatric patients admitted from July 2001 to June 2008 in closed wards of the Department of Psychiatry at the Erenkoy Psychiatric and Neurological Disorders Hospital. Controls were obtained among volunteers. A number of 100 OCD patients and 50 healthy controls were enrolled in to study. The diagnosis was made using Structured Clinical Interview for DSM-IV (SCID-I) (First *et al*., 1997; Ozkurkcugil *et al*., 1999).

The inclusion criteria for OCD patients are as follows: subjects with neurological diseases that may affect neuropsychological status were not included in the patient population. People with alcohol and psychoactive drug use were not included in the patient group.

Our healthy group was formed according to the criteria of no medical or psychiatric history, no alcohol and drug use, no head injury, and no familial predisposition for any psychiatric disease. Our volunteers, who formed the OCD and healthy groups, who participated in the study with their consent, were recruited from the homogeneous group residing in the Marmara region.

### Biochemical parameters

#### Determination of IFN γ levels

High Sensitive ELISA Kit was used for determining IFN γ levels in serum. In this study, prepared standard (10, 5, 2.50, 1.25, 0.63, 0.31, and 0.16 pg/ml) concentrations and serum samples were placed in antibody-coated wells and then studied according to instructions of the kit manufacturer (BMS228HS, Invitrogen Thermo Fischer Scientific, Germany). IFN γ levels were determined as pg/ml. The sensitivity of the IFN γ kit was 0.06 pg/ml (Fluhr *et al*., 2011).

#### Measurement of thiobarbituric acid reactive substances (TBARS) levels

TBARS levels were studied using with colorimetric kit in serum samples (E-BC-K298-M, Elabscience, USA) (Zhou *et al*., 2020). The serum samples and prepared standards were placed in glass tubes containing 2-TBA and acetic acid and incubated at 100 °C in a water bath for 1 h. After incubation, the tubes were allowed to cool and then centrifuged at 1600 g for 10 min. The supernatants were separated and read absorbances at 532 nm. The TBARS concentrations were calculated as umol/L from the curve by obtaining from the standards. TBARS kit has 0.85 umol/L sensitivity.

### Measurement of glutathione peroxidase (GSH-Px) activity

GSH-Px activity was studied by modifying the method of Lawrence and Burke in 1976 (Lawrence & Burk, 1976). In this method, glutathione peroxidase activity is determined by two consecutive reactions. While H_2_O_2_ or organic hydroperoxides (ROOH) in the sample are reduced by the effect of GSH-Px, the GSH turns into oxidised GSH (GSSG). Following the second reaction, GSSG is reduced to GSH with the oxidation of NADPH to NADP^+^ with the effect of GSH-reductase (GSH-R). This conversion is monitored as a decrease in absorbance at 340 nm at 37 °C. The results were presented as nmol/L using the extinction coefficient of NADPH, 6.22 × 10^3^ M − 1× cm^−1^.

### Measurement of tryptophan/kynurenine levels in serum by HPLC

Tryptophan and kynurenine levels were measured in serum samples by the HPLC method. In our study, a C18 (250 × 4.6 mm id; 5 µm) column was used for the solid phase, and Xiang’s method was applied by modified (Xiang *et al*., 2010). Serum samples were treated with an equal volume of perchloric acid and after centrifuging, taken with clear supernatants for analysis in HPLC.

The flow rate of the device was adjusted to 1.5 ml/min, and then, 20 µl of the standard mix was taken with the device injector and loaded into the device and fluorescence detector; kynurenine was read at 365–480 nm and tryptophan at 254–404 nm.

### Genotyping

DNA was extracted with the salting-out method from peripheral blood (Miller *et al*., 1988). Genotyping was performed with polymerase chain reaction (PCR) using forward and reverse primers as follows; F: 5’-TTA GCT GAC CCA CCG TCT CT-3’ and R: 5’-GCC CAT CTG GAT TCC ATT C-3’; for AhR rs10249788, and F: 5’-CAG TGA AGA GGT GTA GCC GCT-3’ and R: 5’-TAG GAG TCT TGT CTC ATG CCT-3’ for CYP1A1 rs4646903, and following restriction fragment length polymorphism (RFLP) was applied.

After PCR processes, amplified DNA products were restricted with BbsI and MspI restriction endonuclease enzymes for AhR rs10249788 and CYP1A1 rs4646903 polymorphisms, respectively. The restricted DNA samples were electrophoresed in 2.5% Agarose gel electrophoresis with loading buffer with DNA size marker at 130 V for 25 min, visualised in a UV transilluminator.

For determining AhR rs10249788, there were bands as follows: cut in base pairs (bp) with C allele (187 bp and 18 bp) and uncut T allele (205 bp). For determining CYP1A1 rs4646903, cut T (270 bp,130 bp) and uncut C alleles (400 bp). Genotypes were evaluated by two separate individuals (Hayashi *et al*., 1991; Wang *et al*., 2012).

### Statistical analyses

Statistical analyses were performed with SPSS version 21. The chi-square test used categorical data for the evaluation of genotyping, gender, smoking, alcoholism, and obsession, compulsion types. Student’s t-test was applied for parametric, and the Mann–Whitney U-test was used for nonparametric distributed data. Pearson’s correlation test assessed the relation between serum cytokines, oxidants, and antioxidants parameters in the OCD group. Sensitivity and 1-specificity curves for IDO activity in OCD and control groups were calculated with receiver operational characteristics (ROC) and area under the curve (AUC) link from https://epitools.ausvet.com.au/roccurves (Greiner *et al*., 2000). The significance level was accepted as *p* < 0.05.

## Results

### Demographic results

Table 1 presents the demographic characteristics of 100 OCD patients and 50 healthy controls related to age, gender, smoking, and alcohol use and also shows disease onset age, types of obsession and compulsion, and suicide in the OCD group. A 59% of the OCD patients and 70% of the control group were women (*p* > 0.05). The mean age of the OCD patients was 39 ± 11.43, and the mean age of the controls was 36.52 ± 10.74. There were no significant differences between the groups in terms of smoking and alcohol use, age, and gender (*p* > 0.05).

**Table 1.** Demographic characteristics related to OCD and control groups

		OCD (*n* = 100)	Controls (*n* = 50)	*p*-value
Gender	Female	59 (0.59)	35 (0.70)	*p* > 0.05
	Male	41 (0.41)	15 (0.30)	
Age (year)*		39 ± 11.43	36.52 ± 10.74	*p* > 0.05
Smoking	Yes	36 (0.36)	17 (0.34)	*p* > 0.05
	No	64 (0.64)	33 (0.66)	
Alcohol	Yes	97 (0.97)	49 (0.98)	*p* > 0.05
	No	3 (0.03)	1 (0.02)	
Disease onset age (year)*		26.18 ± 10.59	–	
Disease duration (year)*		13.29 ± 10.71	–	
YBOSC scale		23.17 ± 8.83	–	
Obsession	Aggression	35 (0.35)	–	
	Contamination	34 (0.34)	–	
	Sexual	34 (0.34)	–	
	Hoarding	79 (0.79)	–	
	Religious	50 (0.50)	–	
	Symmetry	38 (0.38)	–	
	Somatic	74 (0.74)	–	
	Other	61 (0.61)	–	
Compulsion	Cleaning	32 (0.32)	–	
	Checking	23 (0.23)	–	
	Repetitive Ritual	67 (0.67)	–	
	Counting	62 (0.62)	–	
	Ordering	86 (0.86)	–	
	Other	85 (0.85)	–	
Suicide attemption	Yes	81 (0.81)	–	
	No	19 (0.19)	–	

*Mean ± Standard Deviation; OCD: Obsessive-compulsive disorder; YBOSC: Yale Brown Obsessive-Compulsive Scale.

### Biochemical analyses

In biochemical experiments with 100 patients and 50 controls, IFN γ, tryptophan, kynurenine, GSH-Px, and TBARS parameters were studied in serum samples. Also, IDO activity was by the ratio of kynurenine to tryptophan as IDO activity in serum. According to the results, while IFN γ and tryptophan levels were found to be significantly lower in OCD (*p* < 0.001), TBARS levels were found to be significantly higher in patients with OCD than those of controls (*p* < 0.001).

**Figure 1.**
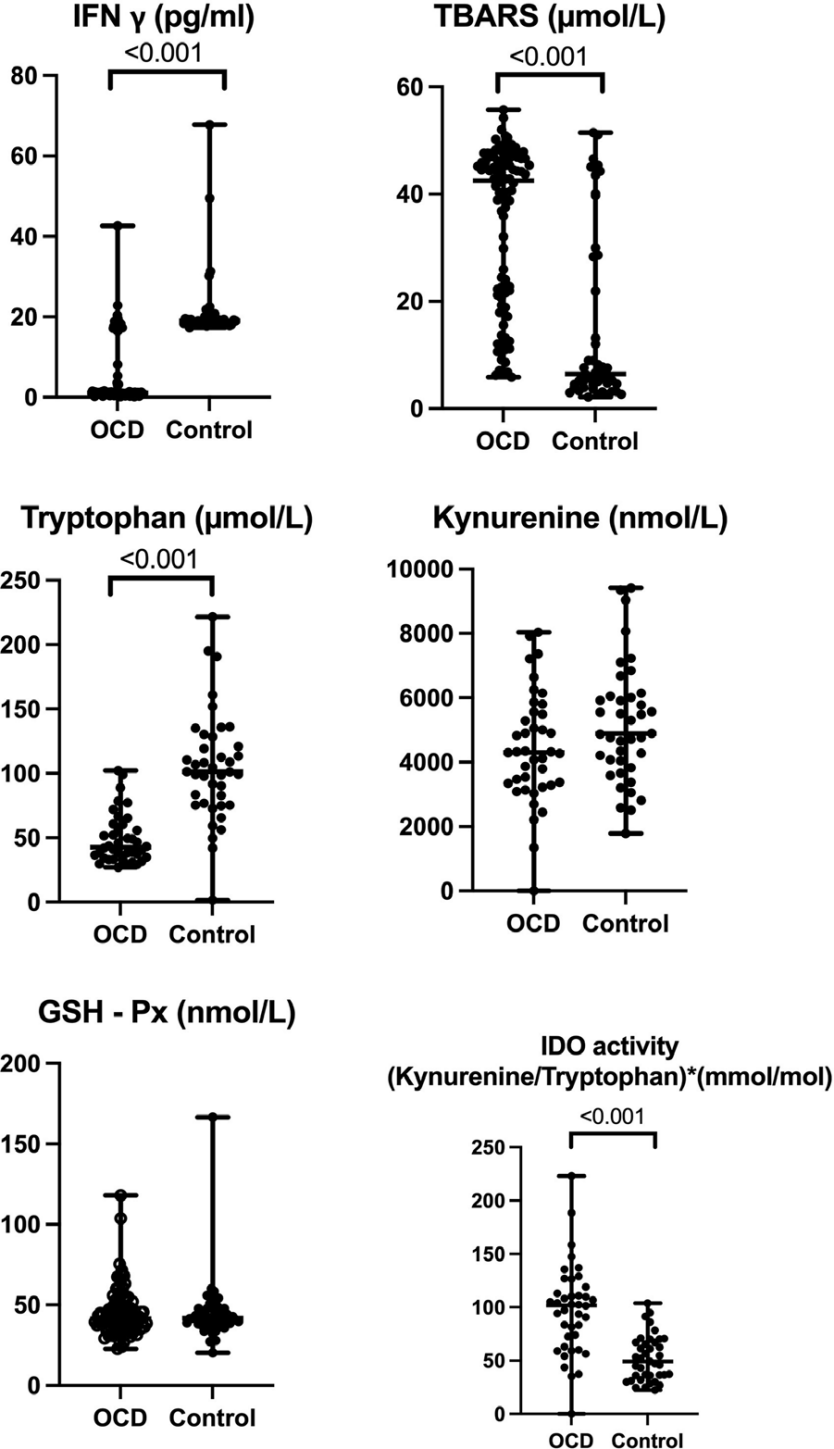
Biochemical characteristics related to both study groups.

No significant differences were found in kynurenine and GSH-Px levels (*p* = 0.108, *p* = 0.83, respectively) (Fig. 1).

When Pearson’s correlation analysis was performed within the OCD patient group, there was a significant direct correlation between IFN γ and TBARS (*r* = 0.220 *p* = 0.028) and GSH-Px (*r* = 0.232 *p* = 0.020). The relationship between serum tryptophan and kynurenine levels was found to be a direct and significant correlation (*r* = 0.388 *p* = 0.013). Also, IDO activity was correlated with the levels of kynurenine (*r* = 0.547, *p* = 0.000) and tryptophan (*r* = −0.513 *p* = 0.001).

**Figure 2.**
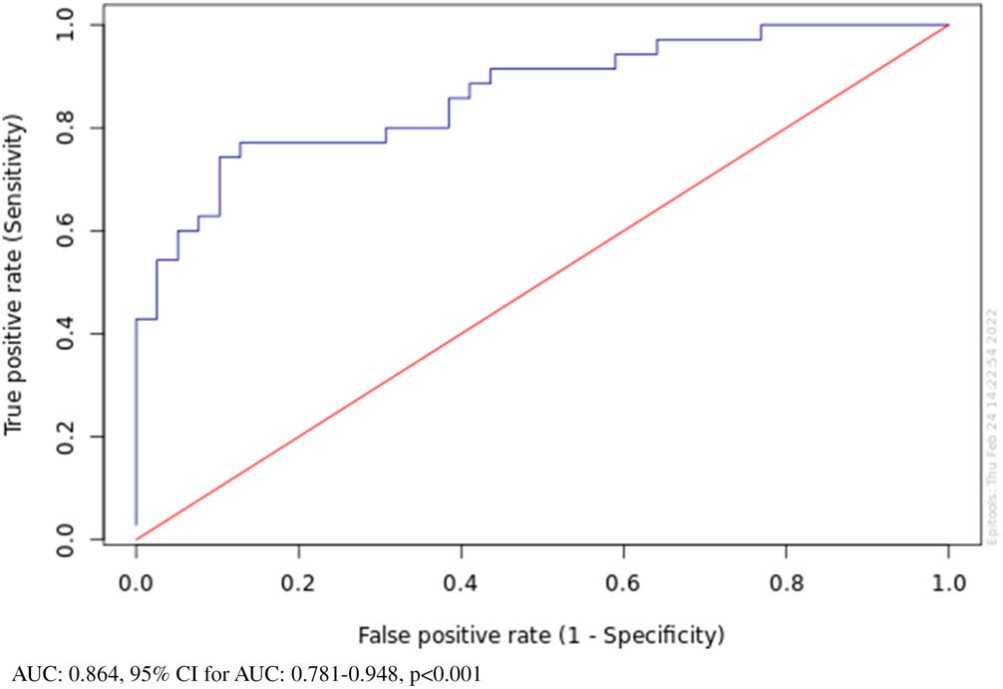
ROC analysis in OCD and control groups according to IDO activity.

The ROC results corresponding to the IDO activity of the OCD and control groups are presented in Fig. 2, where the curves of groups are compared (AUC = 0.864, 95% CI = 0.781–0.948, *p* < 0.001) (Fig. 2).

### Genotype analyses

**Figure 3.**
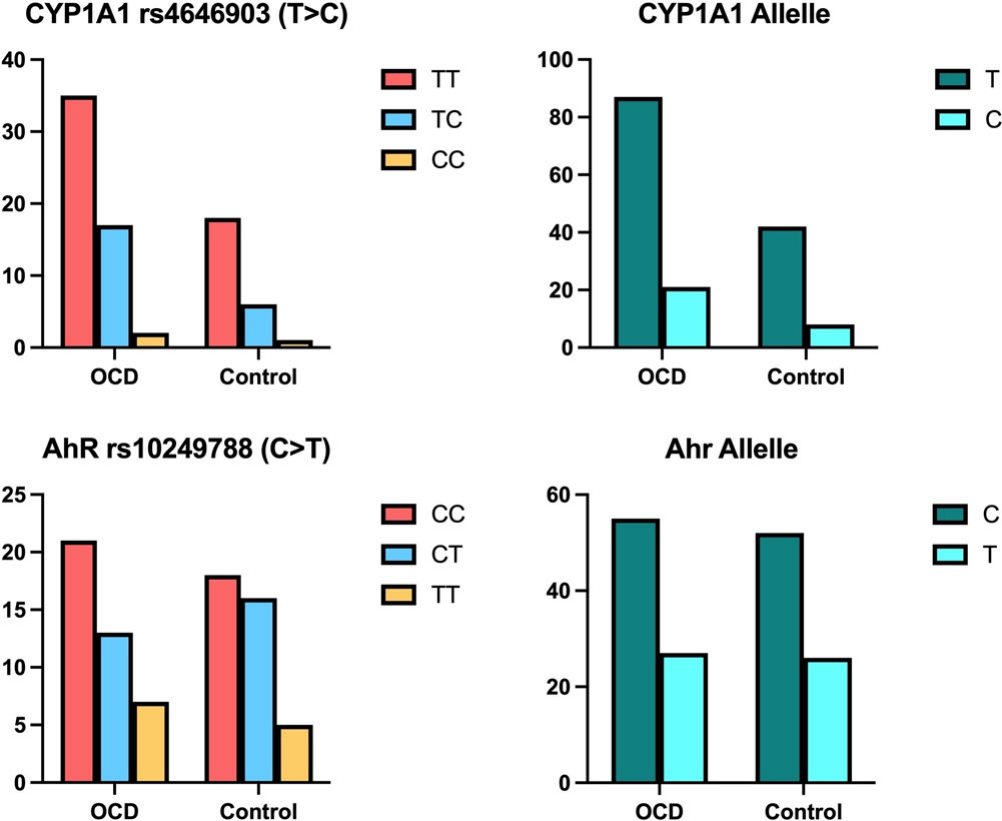
Distributions of CYP1A1 rs4646903 (T > C) and AhR rs10249788 (C > T) genotypes and alleles in both OCD and control groups.

The distributions of AhR rs10249788 (C > T) and CYP1A1 rs4646903 (T > C) genotypes and alleles in OCD and control groups are shown in Fig. 3. No significant differences were observed between the OCD and control groups in terms of genotype and allele frequencies in the CYP1A1 rs4646903 and AhR rs10249788 (Fig. 3).

**Figure 4.**
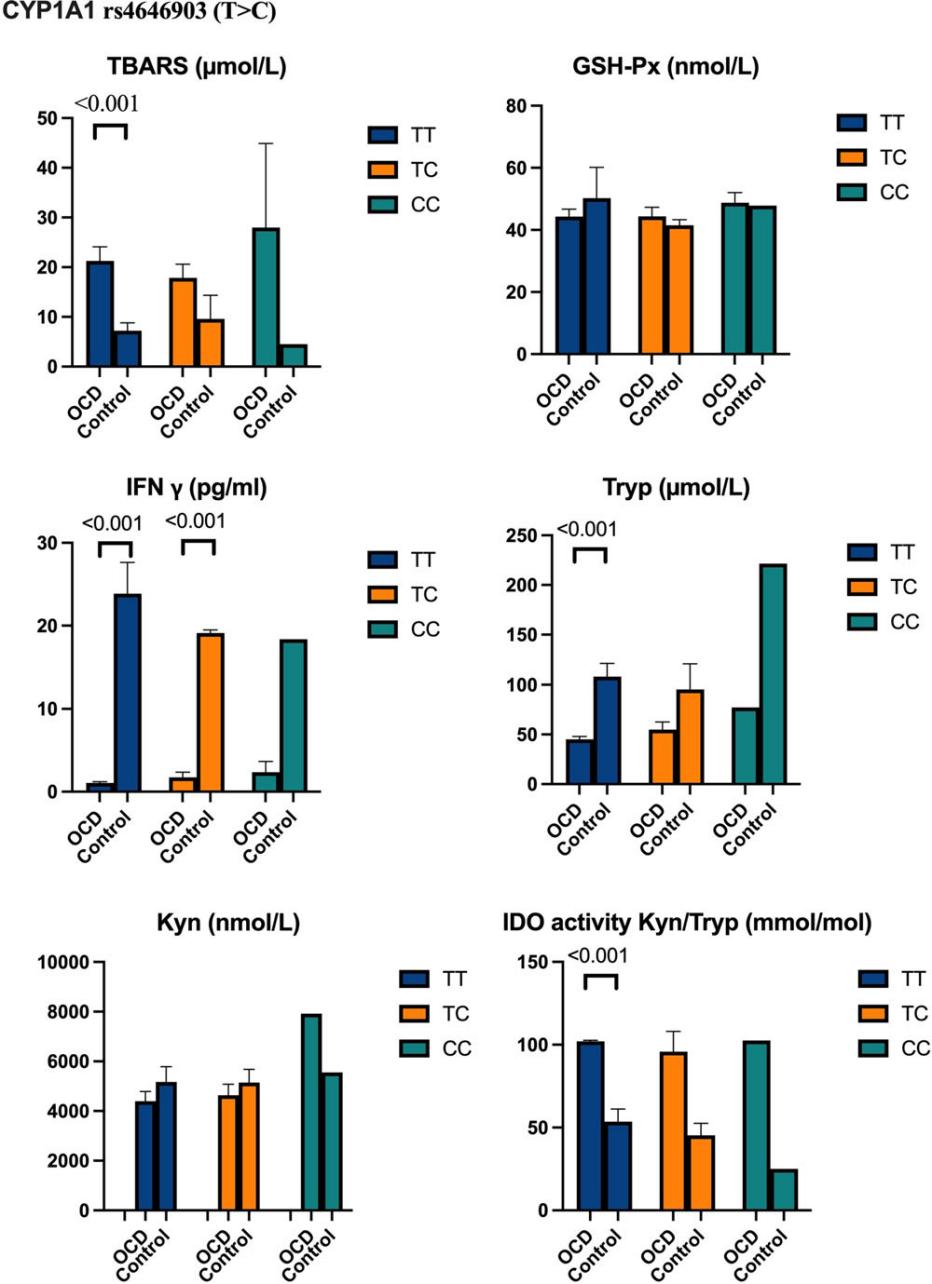
Biochemical results in OCD and control groups according to CYP1A1 rs4646903 (T > C) genotype distributions.

**Figure 5.**
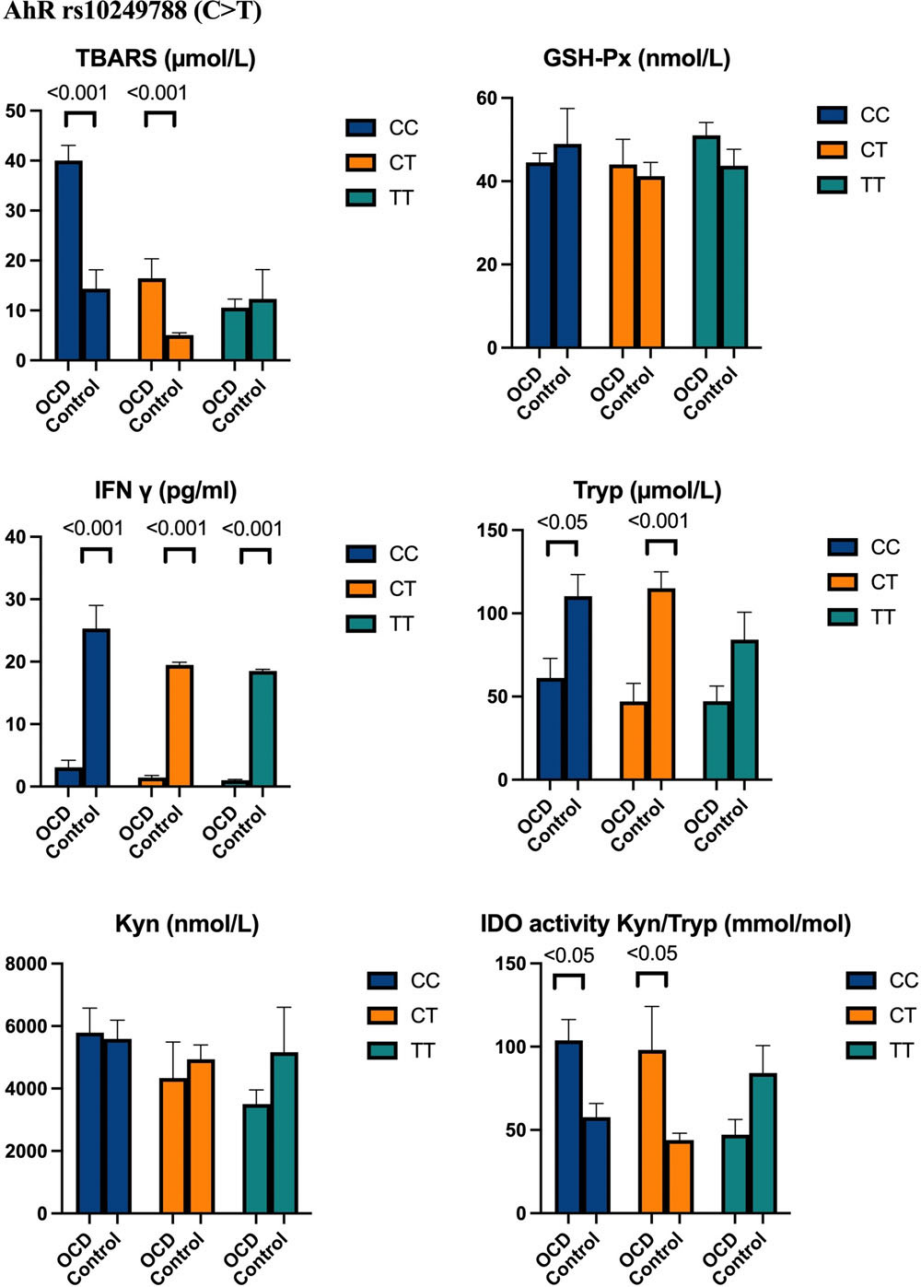
Biochemical results in OCD and control groups according to AhR rs10249788 (C > T) genotype distributions.

We analysed the CYP1A1 rs464903 and AhR rs10249788 genotypes and their correlations with biochemical parameters (Figs. 4 and 5). In Figs. 4 and 5, there are no show biochemical results that belong to allele distributions between the OCD and control groups.

We found that TBARS levels and IDO activities were significantly higher in the OCD group carrying among CYP1A1 TT genotype (*p* < 0.001, for both), T (*p* < 0.001, for both), and C alleles (*p* < 0.001, *p* < 0.05, respectively) than those in the control group. Meanwhile, it was found significant decrements in IFN γ levels in those carrying CYP1A1 TT, TC genotypes (*p* < 0.001, for both genotypes), and T allele (*p* < 0.001) in the OCD group compared to those of the controls.

In the OCD group according to those of controls, tryptophan values were determined to decrease significantly in CYP1A1 TT genotype (*p* < 0.001), T (*p* < 0.001), and C alleles (*p* < 0.05). There were to observe no significant differences in kynurenine and the activity of GSH-Px in those carrying CYP1A1 T > C genotype and alleles in OCD and control groups (Fig. 4).

In the OCD group, TBARS levels and IDO activity were found to be significantly higher in those carrying AhR CC (*p* < 0.001, *p* < 0.05, respectively) and CT genotypes (*p* < 0.001, *p* < 0.05, respectively), and C (*p* < 0.001, for both) and T allele (*p* < 0.001, for both) compared to controls (Fig. 5).

IFN γ levels were found to decrease significantly in those carrying AhR CC (*p* < 0.05), CT (*p* < 0.001), TT genotypes (*p* < 0.001), and C, T alleles (*p* < 0.001, for both alleles) in OCD compared to those in controls. In another hand, tryptophan values were decrements in those carrying AhR CC (*p* < 0.05), CT genotypes (*p* < 0.001), and C, T alleles (*p* < 0.001, for both alleles) in OCD compared to those of controls. There were no significances in both kynurenine and GSH-Px activity in those carrying AhR genotypes and alleles (Fig. 5).

## Discussion

In our study, the effects of inflammation and oxidative stress on the KP in OCD were examined for the first time. Another aim of our study was to research the effects of AhR and CYP1A1 gene variants on OCD and the effects of inflammation and oxidative stress parameters on OCD through these gene variants.

The KP, which is important in the development of neuropsychiatric diseases, is also of great importance because it is under the regulatory influence of glutamatergic neurotransmission and immune effects (Savitz, 2020). In the KP, the first step in the formation of kynurenine via tryptophan is the IDO enzyme, which is rate-limiting and is directly affected by inflammation and oxidative stress factors (Geng & Liu, 2014; Mor *et al*., 2021).

In our study, we found that tryptophan levels were significantly decreased in the OCD group. On the other hand, the decrease in kynurenine levels in the OCD group did not reach a significant difference. The tryptophan/kynurenine ratio is of great importance in terms of showing IDO activity in neuropsychiatric diseases (Quak *et al*., 2014). In our study, it was determined that IDO activity was significantly increased in the OCD group compared to the healthy group. In the OCD group, IDO activity was significantly higher than that of the controls, implying that the power of discrimination has an advanced level according to the ROC analysis. The area under the ROC is used as a comparison scale for the superiority of diagnostic tests. In other words, the IDO test evaluated in the ROC analysis is important in terms of showing both the sensitivity and specificity power of the test as the power to distinguish patients from controls (Hajian-Tilaki, 2013).

In the studies published by Külz *et al.* and Bellodi *et al*., it was reported that the plasma tryptophan level decreased in patients with OCD (Külz *et al*., 2007; Bellodi *et al*., 1997). Our tryptophan level is also consistent with publications (Barr *et al*., 1994; Hood *et al.*, 2017; Cowen *et al*., 1989). IDO activity, in our study, such as the study by Chipelli *et al*. in schizophrenia patients, was found to be high in patients (Chiappelli *et al*., 2016).

In studies, it has been known that the consumption of tryptophan is a rate-determining enzyme in the IDO reaction. The increase in kynurenine levels may cause decrements in brain serotonin levels, and depression and anxiety through the kynurenine pathway (Oxenkrug, 2010). It is known that the IDO enzyme is activated by inflammatory regulators, especially IFN γ, Transforming Growth Factor-β (TGF-β), and IL-6 (Mbongue *et al*., 2015; Salminen, 2022). In our study, we found that IFN γ levels were significantly reduced in the OCD group. The decrease in IFN γ proinflammatory cytokine levels may relate to the increased IDO enzyme and depleted tryptophan levels in OCD (Gasse *et al*., 1997; Wirleitner *et al*., 2003).

There are studies showing that proinflammatory cytokines play a role in the aetiopathogenesis of psychiatric diseases (Pérez-Sánchez *et al.*, 2018). It has been reported that serum IFN γ levels are decreased in patients with major depressive syndrome, it contributes to the formation of immune response in the disease, and it has been reported to be negatively correlated with the severity of depression (Kim *et al*., 2016; Daria *et al*., 2020).

Low IFN γ levels were reported in MDD patients in the meta-analysis study by Köhler *et al*. and in the studies by Himmerich *et al*. (Köhler *et al*., 2017; Himmerich *et al*., 2019).

It was thought that the deterioration of the immune system in OCD might have an effect on oxidative stress markers, and as a result, TBARS or MDA levels, which many study groups examined as the end product of lipid peroxidation, were investigated. In common with our study and other studies, TBARS levels were found to be significantly higher in patients compared to controls (Chakraborty *et al*., 2009). It is known that brain tissue is very sensitive to oxidative stress-related damage due to the richness of its structure in terms of lipid molecules and the use of most of the oxygen in the organism. It has been shown that oxidative stress is closely related to the pathogenesis of diseases such as OCD, substance abuse, bipolar disorder and schizophrenia, which include anxiety and depression (Chakraborty *et al*., 2009; Behl *et al*., 2010; O’Farrell & Harkin, 2015; Madhura, 2015; Shrivastava *et al*., 2017; Maia *et al*., 2019; Mohammadi *et al*., 2021; Baratzadeh *et al*., 2021). In our study, it was found the TBARS levels of the OCD group were significantly higher, similar to the studies in the literature, and there was no significant difference in the antioxidant enzyme GSH-Px activity (Yao *et al*., 1998; Kuloglu *et al*., 2002; Pillai *et al*., 2007; Sarandol *et al*., 2007; Chakraborty *et al*., 2009; Behl *et al*., 2010; Shrivastava *et al*., 2017).

In addition, in a study in which oxidant and antioxidant parameters were examined as total oxidant status (TOS), total antioxidant status (TAS), and oxidative stress index (OSI), it was reported that there was no difference between OCD and healthy groups (Alici *et al*., 2016; Sonkurt *et al*., 2022). As one of the main antioxidant enzymes, GSH-Px is responsible for catalysing the reduction of peroxides in H_2_O_2_. Decreases in GSH-Px enzyme activity were found in patients with schizophrenia, affective disorder, and depression. In our study, we observed that there was no difference in GSH-Px enzyme activities between the OCD and the healthy group, in line with other studies in the literature (Reddy & Yao, 1996; Akyol *et al*., 2002; Srivastava *et al*., 2002; Zhang *et al*., 2010).

When we examine the Pearson correlation results, it shows that IFN γ, which is effective on IDO, is directly related to the oxidative stress parameters TBARS and GSH-Px.

The inverse relationship of IDO activity with the tryptophan molecule at the beginning of the pathway means that tryptophan is consumed upon entry into the pathway. In addition, the direct relationship between IDO activity and kynurenine suggests that it is related to the continuation of the pathway. In our study, the relationship between IDO activity and tryptophan and kynurenine in OCD was found to be compatible with the literature (Gasse *et al*., 1997).

CYP1A1 functions as aromatic hydrocarbon hydroxylase in the first step in the metabolism of polycyclic aromatic hydrocarbons (Nebert *et al*., 1991). In addition, it also functions in the oxidative metabolism of steroids, fatty acids, prostaglandins, and biogenic amines (Kristensen *et al*., 2001). There was no study was found on CYP1A1 gene polymorphic regions in psychiatric diseases. The study on the CYP1A1 m1 polymorphic point, which we examined in the OCD group, is the first study both in our Turkish society and in the literature. In our study, no significant difference was observed between the OCD and healthy groups regarding the CYP1A1 polymorphic region. The MspI-m1 mutation that we examined in our study shares the same region with the m3 mutation in the 3’-flanking region. It has been reported that the functional result of the CYP1A1 m1 mutation increases the inducibility of the gene compared to the wild allele (Crofts *et al*., 1994).

It has been determined that CYP1A1 m1 and adjacent m2 mutations show differences in different ethnic groups. It was found that the frequency of the mutant allele was high in Far East Asian countries, whereas it was found at a low rate in the white race (Kawajiri *et al*., 1990).

AhR interacts with CYP1A1, and there are studies investigating the function of AhR on CYP1A1 (Kiyohara *et al*., 1996; Shimizu *et al*., 2000).

Studies on AhR gene polymorphic regions have been conducted in groups such as endometriosis and infertility, and it has been thought that AhR Arg554Lys and Val189Val may be potential polymorphic regions (Kennedy, 1999; Zheng *et al*., 2015). Studies on autism have reported that these two polymorphic regions may contribute to the phenotype of the disease (Fujisawa *et al*., 2016).

In 2016, Rajendran and Janakarajan conducted studies on ARNT in bipolar disease and examined five single-nucleotide polymorphisms (rs2279287, rs1982350, rs7126303, rs969485, and rs2290035), and reported that the rs2279287 region showed significant differences between patients and controls (Rajendran & Janakarajan, 2016).

In a study on depression, it was found that the AhR rs17137566 polymorphism reduced AhR expression in many tissues (Liu *et al*., 2018). In the literature, there is no study related to the AhR rs10249788 region in psychiatric patient groups. In the OCD group, the results obtained from the AhR rs10249788 polymorphism are the first study on this subject.

In our study, we found that CYP1A1 rs4646903 and AhR rs10249788 genetic variants did not differ significantly in our OCD and control groups. When we analysed the tryptophan, kynurenine, IDO activity, TBARS, IFN γ, and GSH-Px activity in serum values of CYP1A1 and AhR according to the genetic variants studied, we found significant differences in the OCD and control groups.

In our study, we determined that carrying TT genotypes and T, C alleles belonging to the CYP1A1 rs4646903, and AhR rs10249788 polymorphic region had a significant effect on TBARS, IDO, IFN γ, and tryptophan levels in the OCD group compared to our healthy group.

From these results, CYP1A1 rs4646903 and AhR rs10249788 polymorphic points were examined in the literature for the first time in OCD; it was determined that it was effective on TBARS, IFN γ, IDO, and tryptophan levels.

As a result of our study performed for the first time in the OCD group, it has been shown that the KP is affected by proinflammatory IFN γ cytokine and oxidative stress, and CYP1A1 rs4646903, AhR rs10249788 genetic variants are effective on the pathway through TBARS and IFN γ and tryptophan.

Our study is the first study in the literature in terms of examining oxidative stress, inflammation, AhR, and CYP1A1 effective both as the KP and on IDO enzyme activity in the OCD group.

The limitation of our study can be considered that we could not reach more volunteers in OCD and control groups. Another limitation of our study is the inability to investigate gene loci with other enzyme and protein levels that may have a role in KP due to the limited availability of resources.

In relation to our study, it is aimed to study the genes expressing other enzymes involved in the serotonin, melatonin pathway, and KP related to OCD in future studies.

## Appendix

AhR: Aryl hydrocarbon receptor
ARNT: Aryl hydrocarbon receptor nuclear translocator
AUC: Area under the curve
DSM-IV: Diagnostic and Statistical Manual of Mental Disorders
ELISA: Enzyme-linked immunosorbent assay
GSH-Px: Glutathione peroxidase
GSH-R: GSH-reductase
HPLC: High-performance liquid chromatography
IDO: Indolamine-2,3-dioxygenase
IFN γ: Interferon gamma
IL-6: Interleukin 6
KYN: Kynurenine
KP: Kynurenine pathway
NADPH: Nicotinamide adenine dinucleotide phosphate
OCD: Obsessive-compulsive disorder
OSI: Oxidative stress index
PCR: Polymerase chain reaction
RFLP: Restriction fragment length polymorphism
ROC: Receiver operational characteristics
ROOH: Organic hydroperoxide
TAS: Total antioxidant status
TBA: Thiobarbituric acid
TBARS: Thiobarbituric acid reactive substances
TGF-β: Transforming growth factor-β
TOS: Total oxidant status
XRE: Xenobiotic responsible element
